# Development of a thoron calibration chamber based on computational fluid dynamics simulation and validation with measurements

**DOI:** 10.1038/s41598-023-40776-4

**Published:** 2023-08-21

**Authors:** Mohammademad Adelikhah, Morteza Imani, Tibor Kovács

**Affiliations:** 1 Institute of Radiochemistry and Radioecology; Research Centre for Biochemical, Environmental and Chemical Engineering, University of Pannonia, Veszprém, 8200 Hungary; 2 Engineering Department, Shahid Beheshti University, Tehran, Iran

**Keywords:** Environmental chemistry, Environmental impact

## Abstract

Recently, interest in measuring the concentration of ^220^Rn in air has increased greatly following the development of standards and the calibration of monitoring instruments. In this study, a ^220^Rn calibration chamber was designed and developed at the Institute of Radiochemistry and Radioecology (RRI) based on the computational fluid dynamics (CFD) method implemented in ANSYS Fluent 2020 R1 code at the University of Pannonia in Hungary. The behavior of ^220^Rn and its spatial distribution inside the ^220^Rn calibration chamber at RRI were investigated at different flow rates. The ^220^Rn concentration was close to homogeneous under higher flow regimes due to thorough mixing of the gas inside the chamber. Predictions based on CFD simulations were compared with experimentally measured transmission factors (C_out_/C_in_). The spatial distribution of ^220^Rn was dependent on the flow rate and the positions of the inlet and outlet. Our results clearly demonstrate the suitability of the ^220^Rn calibration chamber at RRI for calibrating monitoring instruments. Furthermore, the CFD-based predictions were in good agreement with the results obtained at higher flow rates using experimental and analytical models according to the relative deviation, with a maximum of approximately 9%.

## Introduction

In recent decades, many studies have investigated the health risk of radon (^222^Rn) and thoron (^220^Rn) as well as their progenies on human health. Humans can be affected internally and externally by exposure to radiation that emanates from building materials due to the inhalation of ^222^Rn and ^220^Rn as well as their short-lived decay products, which can emanate from the ground, building materials, and gamma emitting radionuclides^1–10^. Furthermore, it should be noted that the inhalation of these gases and their airborne progeny account for 52% of the natural background radiation dose in humans^11,12^.

National and international agencies that operate under different directives have been responsible for addressing the health risk associated with indoor ^222^Rn and ^220^Rn. Based on the latest scientific data, the World Health Organization (WHO) proposeda reference level of 100 Bq m^−3^ to minimize health hazards due to indoor ^222^Rn exposure^12^. However, if this level cannot be reached under the prevailing country-specific conditions, the chosen reference level should not exceed 300 Bq m^−3^ which represents approximately 10 mSv year^−1^ according to recent calculations by the International Commission on Radiological Protection (ICRP)^13^. The Environmental Protection Agency (EPA) reported that the maximum “acceptable” level of ^222^Rn is 4.0 pCi l^−1^ (150 Bq m^−3^), but even that level is not “safe”^14^. According to the International Atomic Energy Agency (IAEA), basic safety standards for protection against ionizing radiation, the guidelines specific to indoor ^222^Rn are based on ICRP recommendation 126^15^. The upper reference level of 300 Bq m^−3^ is for ^222^Rn exposures in homes^16^.

Reports by the United Nations Scientific Committee on the Effects of Atomic Radiation (UNSCEAR) provide limited information about ^220^Rn exposure, and ^220^Rn research has never been conducted systematically in indoor environments. Therefore, interest has increased in measuring the activity concentration of ^220^Rn gas (conventionally referred to as thoron) in the air. Precisely estimating indoor ^220^Rn concentrations is critical due to concerns about radiological risk and dose assessments, especially considering that ^222^Rn measurements can be distorted^1–3,17–23^. In particular, Kotrappa and Steck in 2010 developed an electret ionization chamber to measure the activity concentration of ^220^Rn gas, where the sensitivity to ^220^Rn was improved by increasing the inlet area for the gas by making extra holes in the sides of the S-chambers^19^. Zhang et al. used a well-maintained ^220^Rn chamber built on site at Peking University in China to accurately measure the activity concentration of ^220^Rn gas by using a Lucas Scintillation Cell and AB-5 measuring device^3^. Eappen et al. also applied a technique for estimating the activity concentration of ^220^Rn gas using a scintillation cell in 2007^17^. Ismail and Jaafar in 2011 determined the optimal size of a ^220^Rn chamber when using a passive CR-39 nuclear track detector. Moreover, Sorimachi et al. and Kavasi et al. in 2012 used a chamber and a room at the National Institute of Radiological Sciences in Japan to check passive and active monitors, as well as assessing the effects of humidity, wind, and ambient aerosols in the air on their measurements^18,24^.

Therefore, methods have been developed for investigating long-term indoor exposure to ^220^Rn (by passive measurement) as well as calibration methods for measuring the activity concentration of ^220^Rn using solid state nuclear track detectors (SSNTDs). Predicting the exact distribution of the activity concentration of ^220^Rn inside a calibration chamber and then calibrating SSNTDs is essential for conducting better assessments based on measurements. However, technical difficulties related to measurements due to the short half-life of ^220^Rn complicate the development of a suitable ^220^Rn calibration chamber and its validation compared with a ^222^Rn calibration chamber.

**Figure 1:**
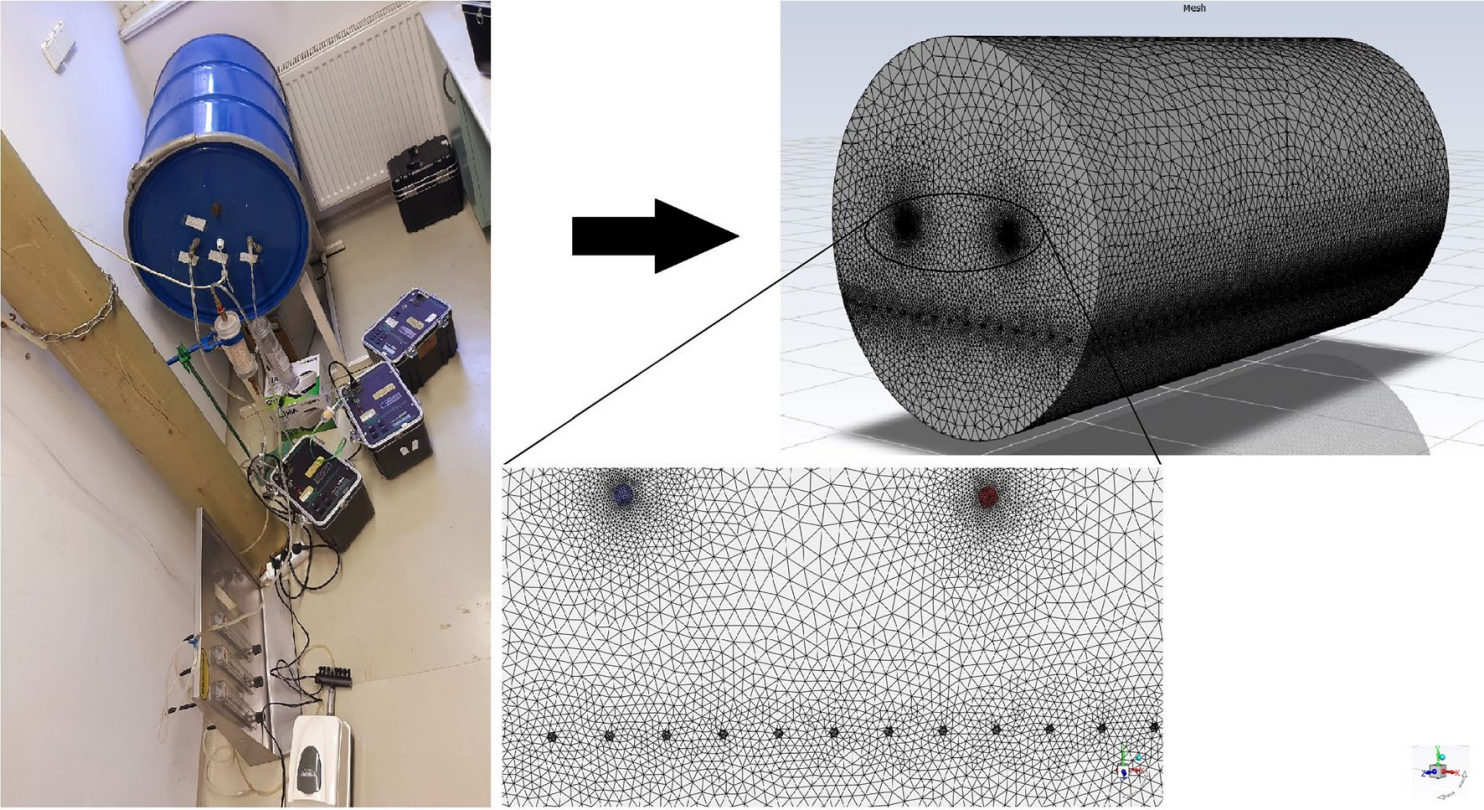
Schematic illustrations of the ^220^Rn calibration chamber and its meshing.

Recently, a ^220^Rn calibration chamber was developed at the Institute of Radiochemistry and Radioecology (RRI) at the University of Pannonia. The distribution of the activity concentration of ^220^Rn inside the chamber can be accurately predicted and simulated by using the computational fluid dynamics (CFD) technique. In previous studies, CFD software was employed as a powerful analytical tool for studying the distributions of ^222^Rn and ^220^Rn inside dwellings^4,21,25–31^. For example, Agarwal et al. used CFD to assess the ^220^Rn distribution in confined volumes in the presence of a forced flow, such as the delay volume used as a mitigation device^21,30^. Therefore, in the present study, the CFD method was used to assess the behavior of ^220^Rn and its spatial distribution inside the ^220^Rn calibration chamber at RRI under different flow rates. The ^220^Rn source is used as a key input parameter in the CFD software (Analysis Systems (ANSYS) Fluent 2020 R1 based on the finite volume method (FVM)) developed and described by Jobbágy and Bety-Denissa (2010)^32^, and Fábián et al. (2017)^33^. Moreover, simulations have been conducted by considering the geometry of the chamber (Fig. 1). In the present study, the transmission factor (ratio of the ^220^Rn concentration at the outlet (C_out_) relative to that at the inlet (C_in_), i.e., C_out_/C_in_) was determined for ^220^Rn in the calibration chamber at RRI under different flow rates. In order to ensure that the calibration chamber operated accurately and to determine the optimum configuration, different configurations were simulated for the positions of the inlet and outlet by using ANSYS Fluent code. Experiments were performed to validate the simulation results and predictions by using the new ^220^Rn calibration chamber experimental setup. Finally, the transmission factors obtained based on CFD simulations, experimental observations, and analytical predictions were compared.

## Models and computational methods

### Geometric model

The geometric model considered in this study was based on the calibration chamber in the laboratory at the RRI, as shown in Fig. 1. The calibration chamber shape was cylindrical with the diameter around 50 cm and length of 100 cm and a volume of 210.5 L. It was made of stainless steel and a lid could be opened to insert instruments for calibration. The temperature and humidity were measured and controlled inside the chamber, and the values were nearly constant at 20–22 °C and approximately 50%, respectively. The chamber had ports for inserting ^220^Rn gas from an external source. Furthermore, unstructured triangular meshes were employed for ANSYS meshing due to their high degree of accuracy, adaptive meshing capabilities, and parallel processing with a minimum volume of 3.92 × 10^−11^ m^3^ using 3,422,491 cells. The convergence criteria i.e., maximum relative difference between two consecutive iteration was taken to be less than 10^−6^.

### CFD simulation approach

CFD software based on the FVM was used to predict and visualize the spatial distribution of ^220^Rn inside the calibration chamber. ANSYS Fluent is an industry leading CFD simulation program with advanced physics modeling capabilities and unequalled accuracy. Many advanced physics models can be implemented using ANSYS Fluent and various fluid phenomena may be analyzed. CFD involves solving a set of nonlinear partial differential equations using numerical methods. Fundamental physical laws are represented by equations that govern fluid flow and related phenomena comprising the conservation of mass, momentum, and energy. The computational domain is enclosed by discretizing and linearizing equations, as well as using relevant boundary conditions (e.g., inlet, outlet, and solid surfaces). The following important assumptions are made: (A) a continuous incompressible airflow; and (B) a homogeneous and uniform temperature distribution inside the chamber. Thus, the steady state internal flow field can be expressed by the continuity and conservation of momentum equations, respectively, as follows^4,25,31^:

1 $$\rho \left(\nabla \cdot {U}_{i}\right)=0$$

2 $$\rho \left(\frac{\partial {U}_{i}}{\partial t}+\nabla \cdot \left({U}_{j}{U}_{i}\right)\right)=-\nabla \cdot P+\nabla .\left({\mu }_{e}{\nabla U}_{i}\right)$$

where *U*_*i*_ and *U*_*j*_ denote the velocity vector (m s^−1^) (*i* and *j* are indices representing the velocity components), *P* represents the pressure (N m^−2^), and *μ*_*e*_ denotes the effective viscosity (N s m^−2^), which can be mathematically expressed as *μ*_*e*_ = *(μ* + *μ*_*t*_*)*, where *μ* and *μ*_*t*_ refer to the dynamic and turbulent viscosity, respectively. The standard k-ε model was used to incorporate the effect of turbulence on the flow field given that it is capable of describing the phenomenon investigated, as shown by many previous studies^4,24,28,31^. In Eqs. (1) and (2), *ρ* represents the density (kg m^−3^). Furthermore, the following equation was used in order to simulate the dispersion of ^220^Rn gas inside the calibration chamber:

3 $$\frac{\partial C}{\partial t}=\nabla .\left({\mathrm{D}}^{*}\nabla C\right)-\nabla .\left(UC\right)-\lambda C$$

where *C* represents the ^220^Rn concentration in the domain volume (Bq m^−3^), D* denotes the effective ^220^Rn diffusion coefficient (m^2^ s^−1^), *U* denotes the mean airflow velocity (m s^−1^), and *λ* is the ^220^Rn decay constant (0.01246 s^−1^). During the simulation, the walls of the calibration chamber were isolated, and the Neumann boundary condition was applied. The main airflow was along the z-axis of the coordinate system used for the simulation.

Initially, to determine the optimum positions of the inlet and outlet as well as the spatial distribution of ^220^Rn inside the calibration chamber, simulations were conducted using different chamber configurations and flow rates (5–100 L min^−1^), as shown in Table 1. Simulations were conducted until convergent results were obtained in each case.

**Table 1:** Different positions of the inlet and outlet in the thoron calibration chamber for CFD simulations.

Chamber configuration	Position coordinates (x, y, z) along the axis (m)	
	Inlet port	Outlet port
I	5, 0, 0	− 5,0, 0
II	10, 0, 0	− 10, 0, 0
III	− 10, − 10, 0	10, − 10, 0
IV	10, 10, 0	− 10, 10, 0
V	15, 0, 0	− 15, 0, 0
VI	10, 0, 0	− 10, 0, 100

### Analytical prediction

**Figure 2:**
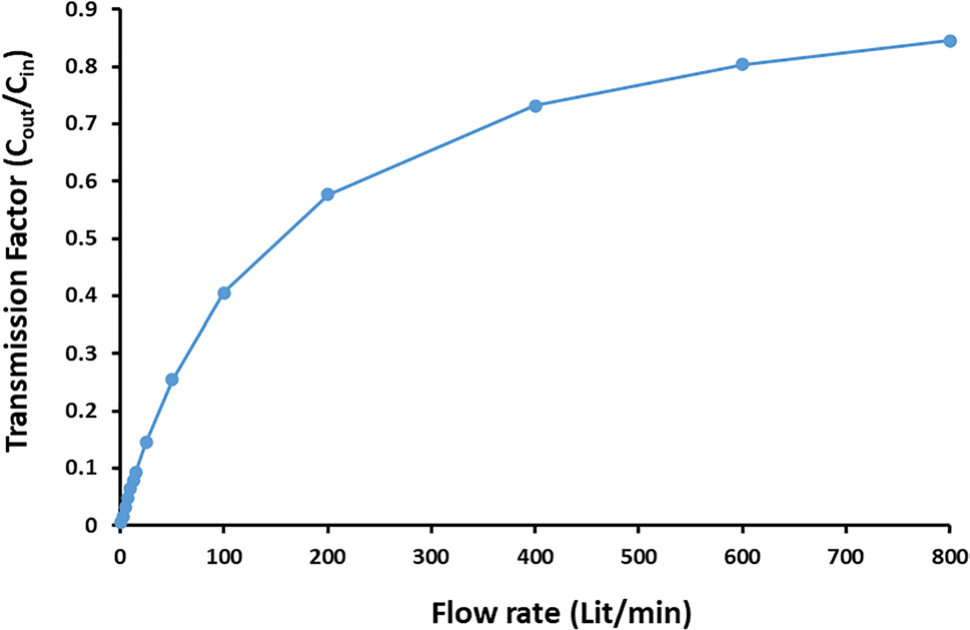
Variations in the transmission factor for ^220^Rn under different flow rates according to the analytical model.

To predict the transmission factor for thoron, the ^220^Rn concentration at the outlet (C_out_) was also estimated analytically using a chamber with a specified volume under a given flow rate and subjected to a particular ^220^Rn concentration at the inlet (C_in_). Therefore, by applying the uniform mixing model based on the assumption that a gas mixes uniformly inside a closed volume, the ^220^Rn concentration at the outlet (C_out_) was calculated according to the following equation^21^:

4 $$\frac{{C}_{out}}{{C}_{in}}=\frac{1}{1+\frac{\lambda V}{Q}}$$

where *C*_*out*_ and *C*_*in*_ denote the ^220^Rn concentrations at the outlet and inlet (Bq m^−3^), respectively, *λ* is the decay constant for ^220^Rn (0.01246 s^−1^), *V* denotes the volume of the closed chamber (m^3^), and *Q* is the volumetric flow rate (m^3^ s^−1^), which was measured by a rotameter in the inlet and applied to calculate the transmission factor for ^220^Rn (C_out_/C_in_). Consequently, by assuming the uniform mixing model, the variations in the transmission factor with the flow rate (Q) is illustrated in Fig. 2. As can be seen from this figure, the transmission factor approached saturation at very high flow rates.

### Configure optimization

In this study, simulation models of several chamber configurations were assessed using the FVM method implemented in ANSYS Fluent CFD code in order to facilitate the design of an initial version of a ^220^Rn calibration chamber for measuring the activity concentration of ^220^Rn and calibrating SSNTDs. Based on the parallel capabilities of meshing and equation solving in ANSYS Fluent, previous CFD simulations have considered the geometry of the chamber but not the tray (to reduce the amount of meshing and the convergence time) by varying the flow rates as well as the positions of the inlet and outlet in order to determine their optimum locations. The use of a tray inside the chamber is for installing the devices and detectors regarding calibrating SSNTDs, intercomparison experiment and so on.

### Experimental setup

**Figure 3:**
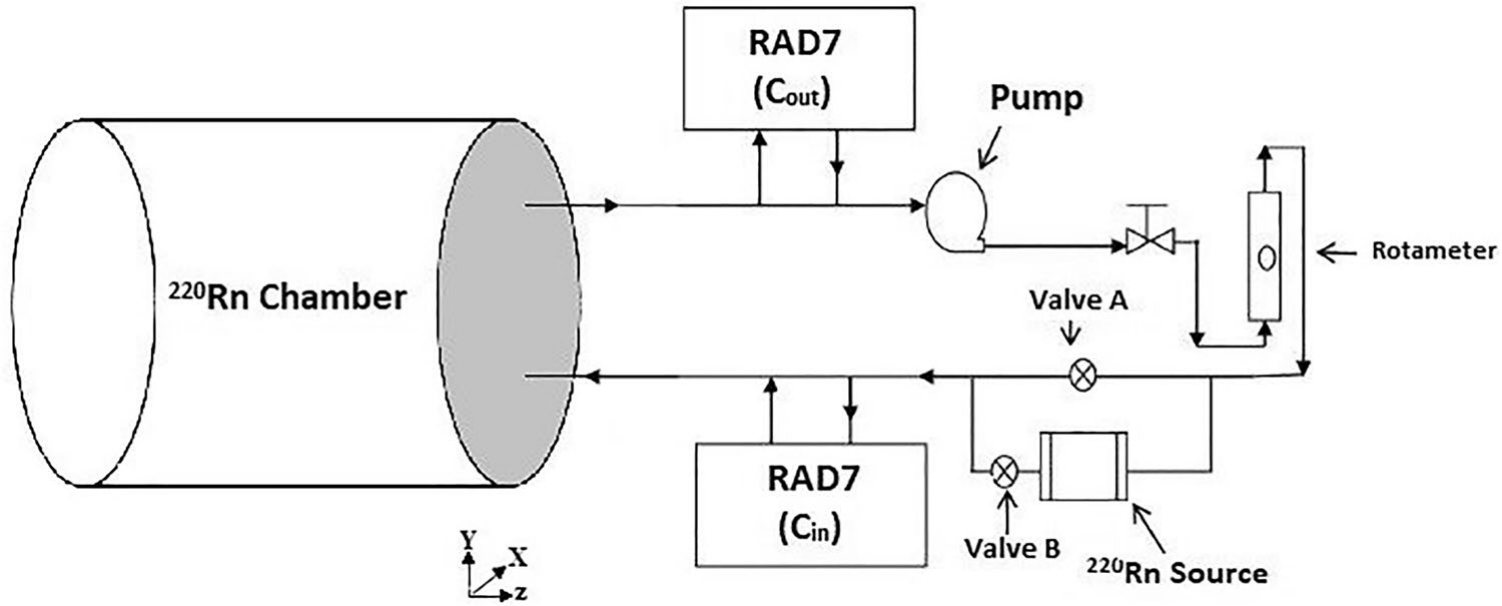
Experimental setup used for measuring ^220^Rn activity concentrations in the ^220^Rn calibration chamber at RRI.

Three different ceramic sources with different thorium contents were available at RRI (Jobbágy and Bety-Denissa, 2010) and previous experiments demonstrated that these sources are suitable for research purposes. Therefore, the investigations in the present study utilized these sources^22,33^, where concentrations of up to 19 kBq m^−3^ could be produced inside the chamber by the different sources. The ceramic sources were produced by Sibelco. In the present study, the source was placed outside the chamber to obtain optimized configuration II according to the simulations. Air passed through a drying column and filter, before it was pumped through the source to enter the chamber (Resun LP-40 air pump, also aquarium tube was used to connect and instruments, source and chamber). Air that exited via the outlet was recirculated through the source to create a closed loop. A schematic diagram illustrating the experimental setup is shown in Fig. 3.

The activity concentrations of ^220^Rn gas were continuously measured at the inlet (C_in_) and outlet (C_out_) by using RAD7 radon/thoron detectors (Durridge, USA). The RAD7 was calibrated by the producer. The measurements were acquired using the “thoron protocol” over a 5 min repeating cycle. The measuring instruments were placed outside the chamber and connected to it by plastic pipes. The half-life of ^220^Rn is less 55.6 s, so measurements of the activity concentration of ^220^Rn gas commenced immediately after sampling. Five sets of experiments were conducted using the optimized chamber configuration and different flow rates to obtain the steady state C_in_ and C_out_ values, before calculating the transmission factor for thoron.

## Results and discussion

### Determination of the optimized configuration

**Figure 4:**
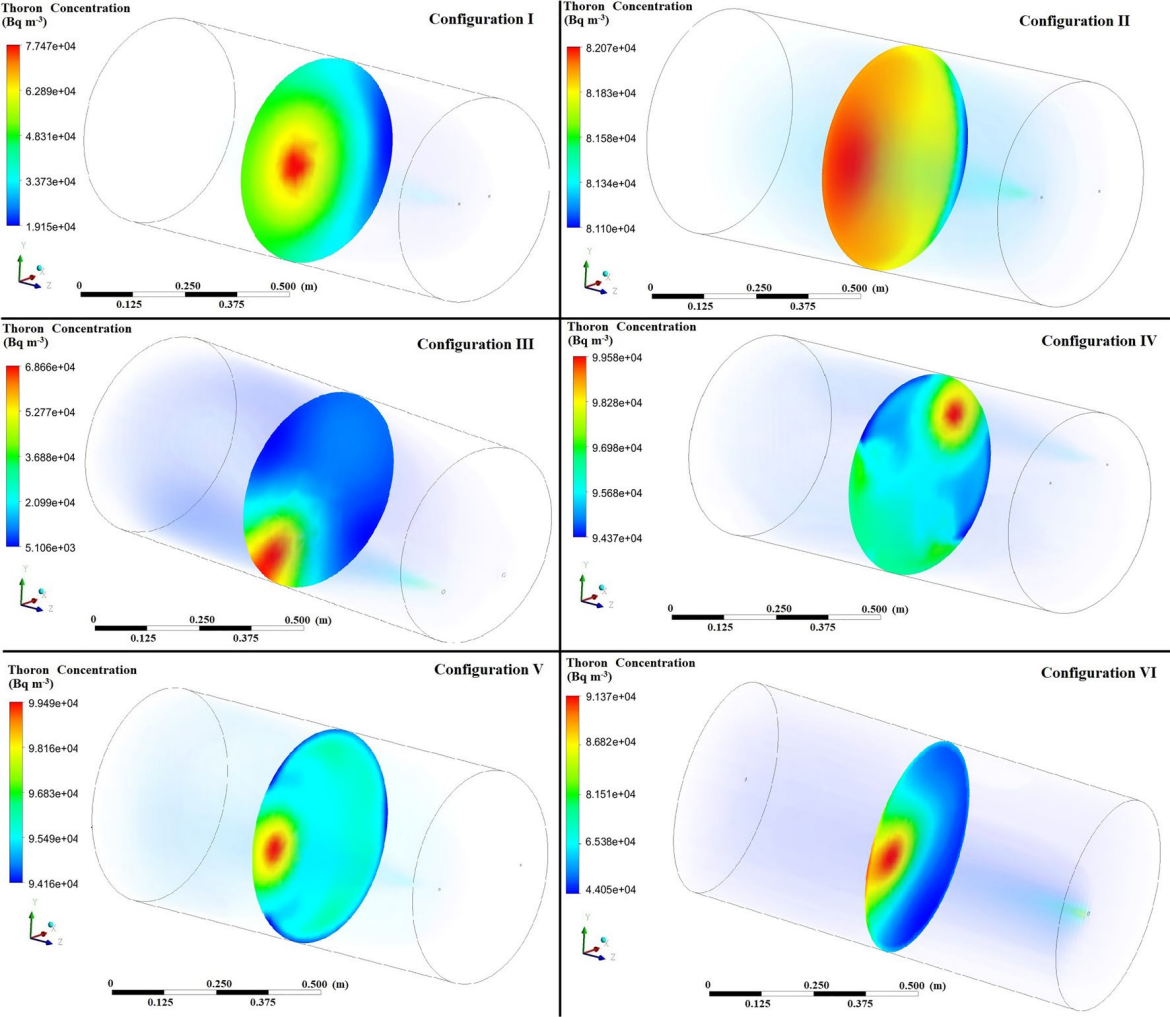
Distribution of ^220^Rn concentration (Bq m^−3^) at the middle of the chamber without tray for different configurations at flow rate of 10 L min^−1^.

Accordingly, Fig. 4 displays the simulated contours of the distribution of ^220^Rn (Bq m^−3^) at the middle of the chamber without tray for different configuration of inlet and outlet with constant airflow velocities of 10 L min^−1^. Moreover, Table 2 shows the ^220^Rn transmission factors simulated based on the distribution of the ^220^Rn concentration inside the chamber for various configurations with flow rates of 5–100 L min^−1^. As expected, the transmission factors were enhanced by higher flow rates under all of the chamber configurations. Configuration II where the inlet and outlet were located symmetrically along the x axis at a distance of 10 cm from the center of the chamber obtained the highest transmission factor. Therefore, these inlet and outlet positions were used in the subsequent CFD simulation and validated based on experimental and analytical results. Table 2 shows that the transmission factors differed for all of the configurations with similar flow rates because changing the configuration affected the airflow profile inside the calibration chamber to modify the ^220^Rn distribution.

**Table 2:** Simulated values of thoron transmission factor (C_out_/C_in_) for various chamber configurations under different flow rates (L min^−1^).

Flow rate (L min^−1^)	Thoron transmission factor (C_out_/C_in_)						Analytical prediction	% Rel. Dev* with Config. II
	Config. I	Config. II	Config. III	Config. IV	Config. V	Config. VI		
5	0.0233	0.0253	0.0240	0.0239	0.0231	0.0248	0.0329	29.62
7.5	0.03534	0.0386	0.0371	0.0373	0.0337	0.0381	0.0486	25.90
10	0.0518	0.0531	0.0512	0.0506	0.0452	0.0523	0.0638	20.16
15	0.0784	0.0816	0.0785	0.0773	0.0737	0.0796	0.0927	13.61
20	0.1012	0.1066	0.1023	0.102	0.1014	0.1050	0.1199	12.47
25	0.1242	0.1351	0.1283	0.1282	0.1274	0.1288	0.1455	7.71
50	0.2164	0.2410	0.2344	0.233	0.2317	0.2331	0.2541	5.44
100	0.3815	0.3931	0.3842	0.3833	0.3813	0.3855	0.4053	3.13

*% Relative deviation between simulation results and uniform mixing model calculated using the following equation:

% Rel. Dev. = $$\frac{\left|\mathrm{Simulation\, result}-\mathrm{ Analytical\, model\, prediction }\right|}{\mathrm{Simulation\, result}}$$.

In order to represent the non-uniformity of a concentration field, coefficient of variation (CoV) of the ^220^Rn concentration is also calculated. Hence, transient variation of CoV for each flow rate and inlet–outlet position were calculated and defined as^34^:

5 $$CoV=\frac{1}{\overline{{C }_{i}}}\sqrt{\frac{\sum_{i=1}^{N}{\left({C}_{i}-\overline{{C }_{i}}\right)}^{2}}{N}}$$

where $\overline{{C }_{i}}$ is the volume averaged concentration 0, ${C}_{i}$ denotes the concentration at each sample point and *N* represents the number of sample points. In the current study, the sample points are taken as all the cells and, therefore, *N* is the number of cells. When the CoV of the distribution becomes less than 10% permanently since the release of gas, distribution is considered uniform. It is a very useful concept to study the transient and spatial behavior of ^222^Rn/^220^Rn concentration in the closed domain. In case of non-uniform mixing, a useful parameter, the mixing time, is used to quantify the time needed to reach a well-mixed state. Table 3 presents the CoV of ^220^Rn concentrations in the chamber for different flow rates and configurations. As can be seen, configuration II shows the lower CoV resulting in the uniformity of gas inside the chamber for further validation step. The value of CoV for configuration number 2 is lower than other geometries, and the difference between this geometry and others is much higher at low flow rates. Also, it is evident that higher flow rate provides remarkably better mixing efficiency.

**Table 3:** CoV of thoron concentrations in the chamber for different flow rates and configurations.

Flow rate (L min^−1^)	Config. I	Config. II	Config. III	Config. IV	Config. V	Config. VI
5	0.4321	0.2797	0.4352	0.4233	0.4111	0.3151
7.5	0.2955	0.2461	0.2935	0.2979	0.3012	0.2786
10	0.2041	0.1937	0.2229	0.2228	0.2247	0.2061
15	0.1655	0.1484	0.1535	0.1506	0.1527	0.1519
20	0.1067	0.1037	0.1147	0.1149	0.1162	0.1105
25	0.0897	0.0890	0.0929	0.0917	0.0933	0.0904
50	0.0490	0.0462	0.0471	0.0471	0.0474	0.0472
100	0.0259	0.0231	0.0237	0.0235	0.0239	0.0233

### Results of CFD simulations

**Figure 5:**
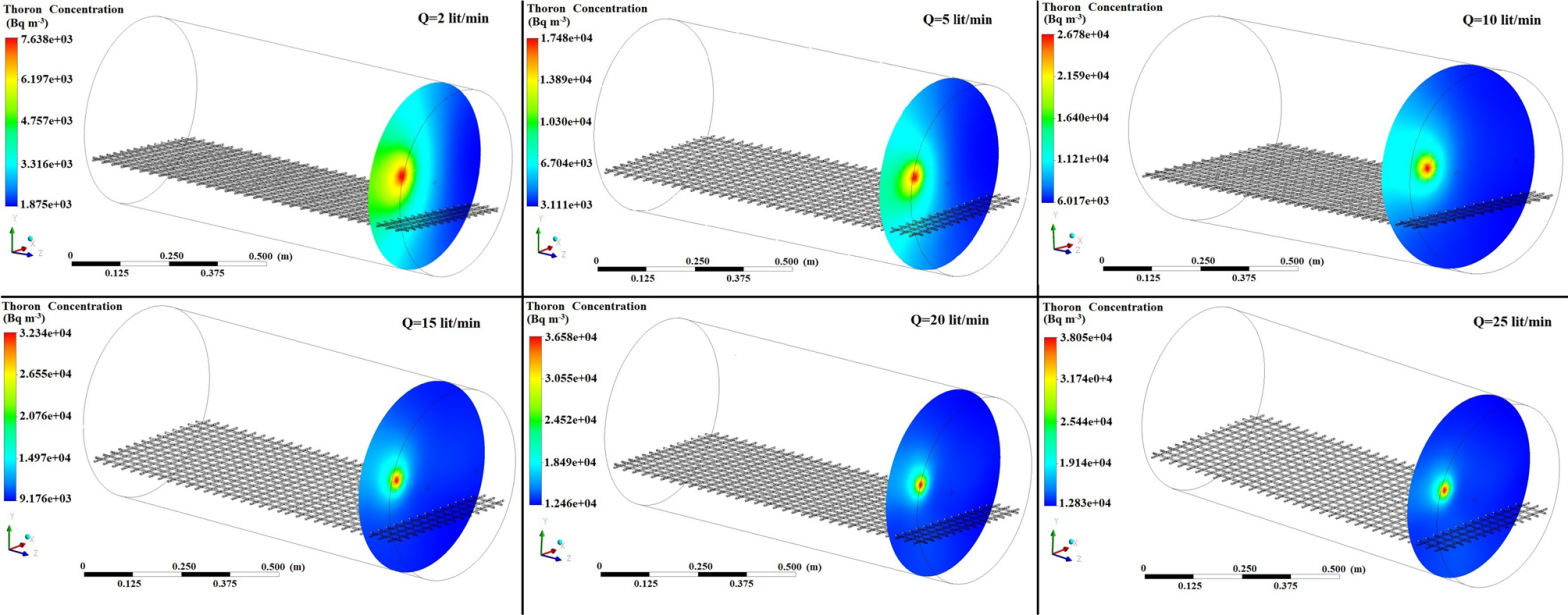
Distribution of ^220^Rn concentration (Bq m^−3^) at Z = 10 cm and different flow rates for optimized chamber configuration II.

**Figure 6:**
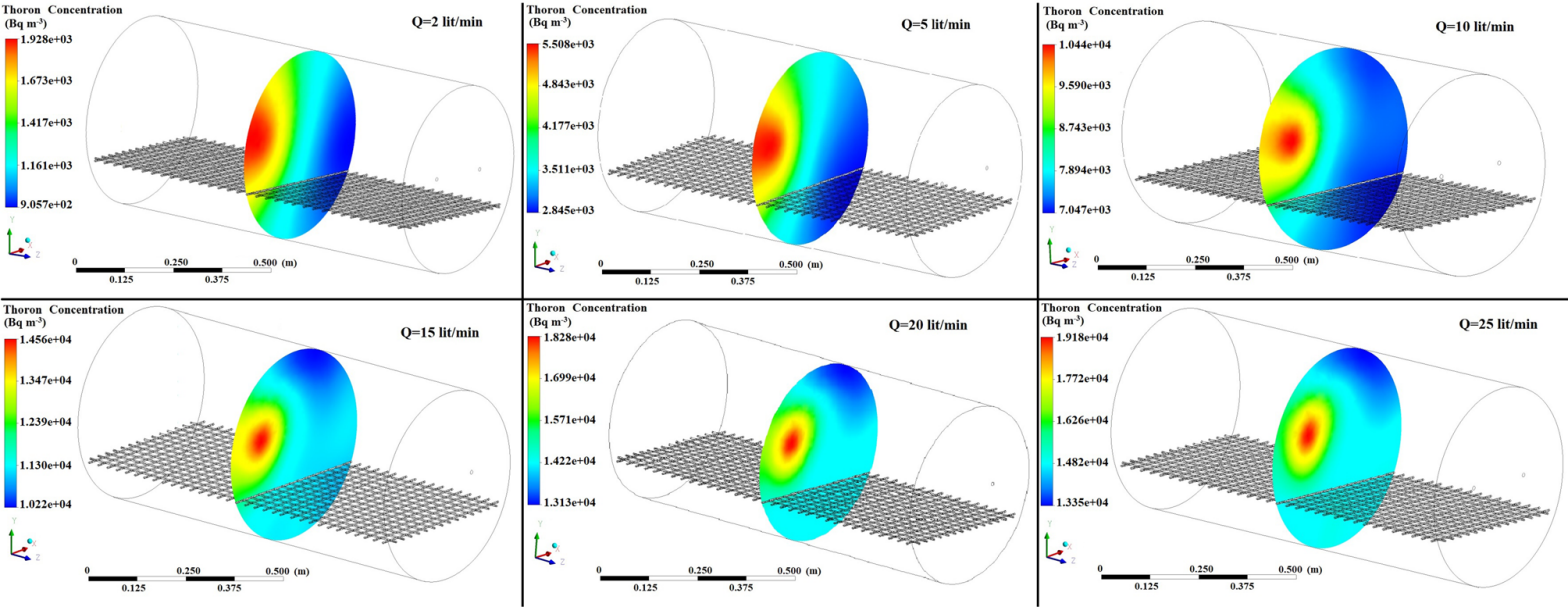
Distribution of ^220^Rn concentration (Bq m^−3^) in the middle of the chamber (Z = 50 cm) and at different flow rates using optimized chamber configuration II.

**Figure 7:**
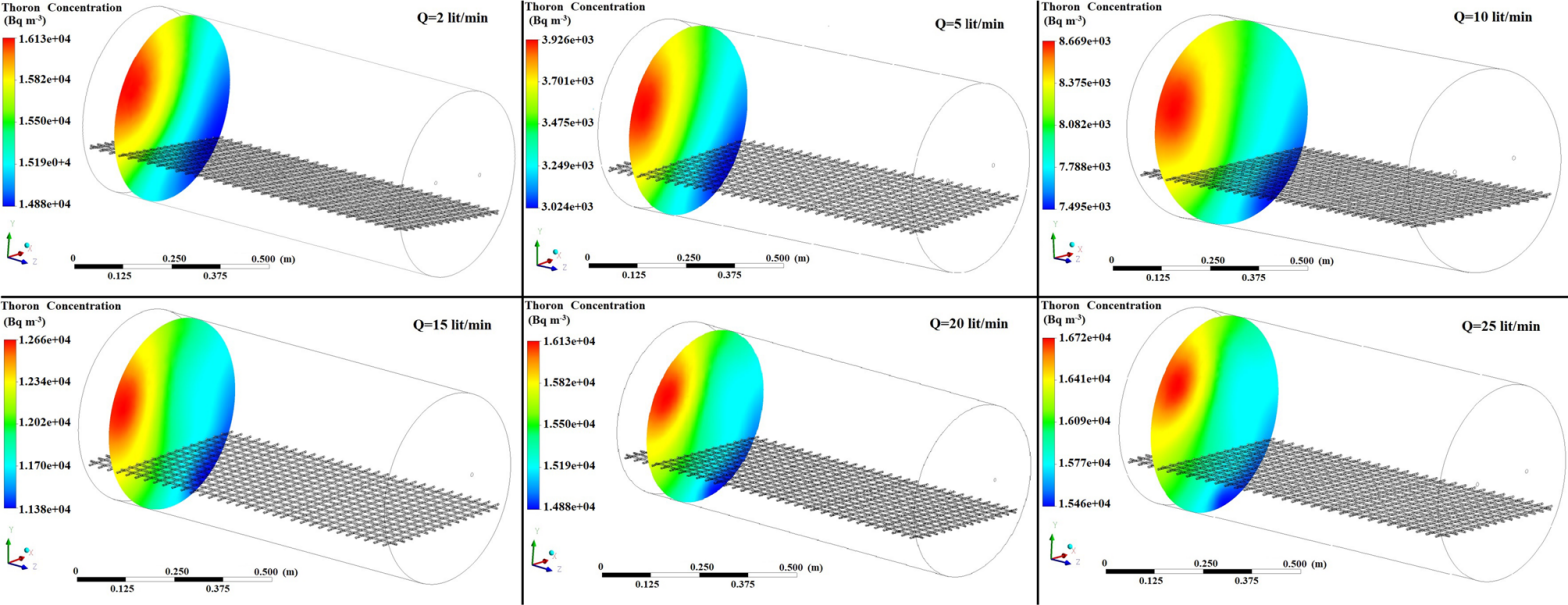
Distribution of ^220^Rn concentration (Bq m^−3^) near the end of the chamber (Z = 90 cm) and at different flow rates using optimized chamber configuration II.

**Figure 8:**
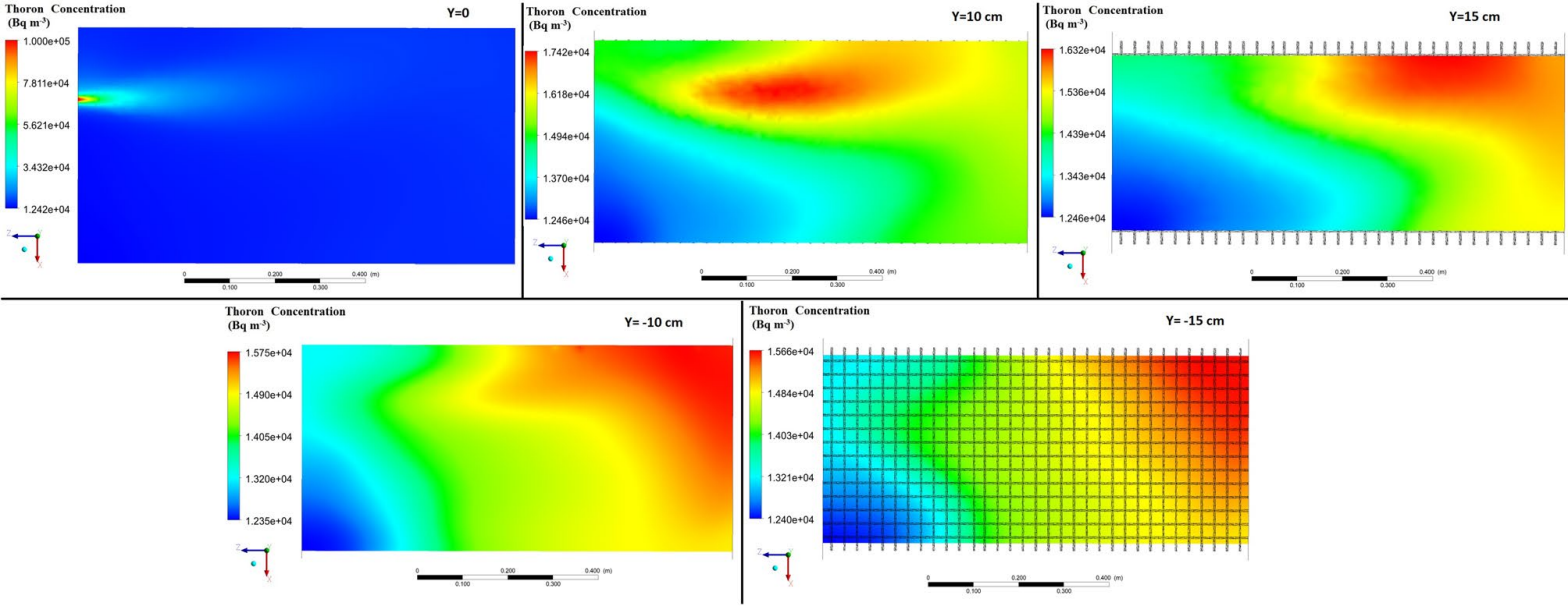
^220^Rn distribution profile (Bq m^−3^) on different horizontal planes inside the chamber at flow rate of 20 L min^−1^ using optimized chamber configuration II.

Figures 5, 6, 7 show the simulated contours of the distribution of ^220^Rn (Bq m^−3^) inside the chamber at different distances from the inlet and various airflow velocities after setting the input parameters under optimized configuration II in the CFD code. The CFD results show that air entered into the calibration chamber as a jet flow and hit the opposite wall before spreading out in all directions, where the concentration was comparatively higher near to the left-hand side wall and corner. Figures 5, 6, 7 show that when the flow rate increased, the fluid hit the end of the chamber with a higher impulse, thereby causing the fluid to spread rapidly. Furthermore, the ^220^Rn concentration tended to be more uniform under higher flow regimes due to thorough mixing of the ^220^Rn gas inside the chamber. Thus, the flow rate and turbulence had important effects on the distribution and mixing of the ^220^Rn gas within the closed volume. To the better understating and visualizing of ^220^Rn distribution uniformity inside the chamber, the ^220^Rn distribution profile on horizontal planes at flow rate of 20 L min^−1^ is also shown in Fig. 8.

**Figure 9:**
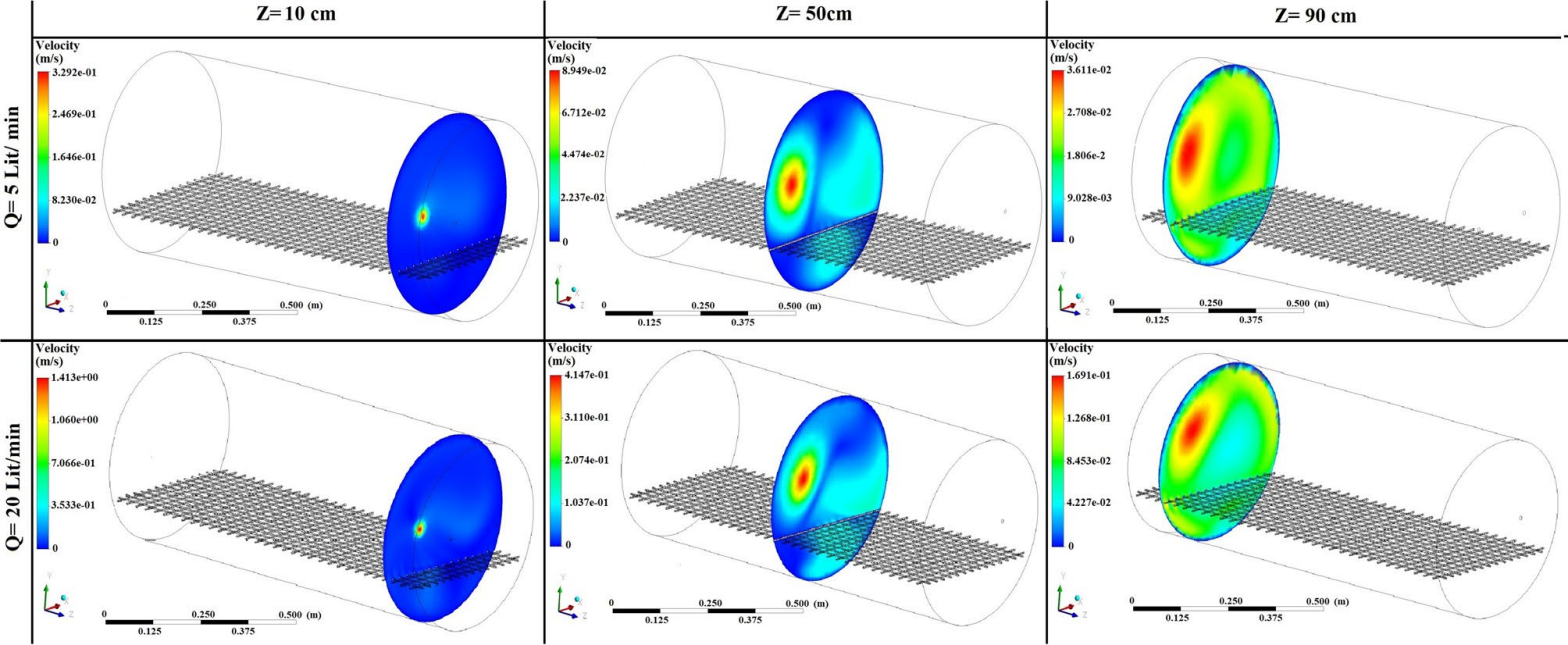
Velocity pattern of air flow inside the chamber at different distances and at two flow rates using optimized chamber configuration II.

Figure 9 shows the velocity patterns of air flow inside the chamber at different distances from the inlet and at flow rates of 5 and 20 L min^−1^ using optimized chamber configuration II. As the airflow rate changed, the distribution of the ^220^Rn concentration inside the calibration chamber varied to affect the transmission factor. It should be mentioned that the inlet is located in the left side, as can be seen in all figures, and that’s why the concentration is higher in left region of the chamber.

### Experimental observations and validation results

Table 4 shows the transmission factors obtained for ^220^Rn (C_out_/C_in_) based on experiments, simulations, and analytical models in the ^220^Rn calibration chamber at RRI under various flow rates of 2–25 L min^−1^ based on optimized configuration II. The CoV factor for simulations was also reported. Typically, radon calibration chambers tend to be used at lower air flows, 2–5 L min^−1^ is very common in the literature, however testing for higher airflows might be interesting for specialist applications with forced air flows, such as mining, and for personal dose monitoring applications testing up to 30–40 L min^−1^ might be necessary^35^. At higher air flows the deposition velocity of the daughter elements might be changed with the airflow^36^, however for SSNTD-s placed in a diffusion chamber (for example CR-39 placed in a RADUET) the signal comes from radon getting into the chamber by diffusion and decaying inside^37^. The air exchange rate of the diffusion chamber should be the determining factor^38^. The air flow might be an issue for the active devices as well, but that can be controlled by the sampling air flow^39^. At the lower flow rate of 2 L min^−1^, the relative deviations between the experimental observations and the simulation results and analytical model predictions were significantly high at 44.74% and 49.93%, respectively. These deviations can mainly be explained by the non-uniformity of the gas inside the chamber which is approved by high value of CoV factor as 0.44. The presence of a tray inside the chamber (for installing the active and passive devices) which intensified the non-uniformity of the ^220^Rn gas, whereas the analytical model is based on the assumption that the gas mixes uniformly inside the closed volume. The deviations reduced after increasing the flow rate, which was expected due to the enhanced mixing caused by the turbulence regime. In particular, the relative deviations were calculated as 18% and 8% at flow rates of 5 L min^−1^ and 25 L min^−1^, respectively. Furthermore, after computing the percentage differences between the estimated results according to ANSYS Fluent and the mixing model calculations, the differences were determined as 31% and 9% at flow rates of 5 L min^−1^ and 25 L min^−1^, respectively. In the case of CoV, in flow rate of 20 L min^−1^ and more, the gas goes toward the uniformity. Based on Table 4, it implies that the well-mixed assumption cannot always be applied to the flow rates of lower than 20 L min^−1^.

**Table 4:** Transmission factors obtained for thoron based on experiments, simulations, and analytical models.

Flow rate (L min^−1^)	Thoron transmission factor (C_out_/C_in_)		Analytical method	Rel. Dev* (%)	Rel. Dev** (%)	Rel. Dev*** (%)	Rel. SD**** (%)	CoV
	Config. II with tray	Experimental						
2	0.0089	0.0062	0.0134	44.74	49.93	116.1	15	0.448
3	0.0142	0.0109	0.0201	30.13	41.11	84.4	14	0.423
4	0.0203	0.0158	0.0265	28.40	30.80	67.72	16	0.409
5	0.0250	0.0212	0.0329	18.22	31.31	55.18	13	0.392
7.5	0.0369	0.0317	0.0486	16.16	31.78	53.31	14	0.259
10	0.0524	0.0441	0.0638	18.98	21.75	44.67	19	0.188
12.5	0.0651	0.0559	0.0785	16.57	20.44	40.42	13	0.166
15	0.0808	0.0708	0.0927	14.14	14.79	30.93	16	0.124
20	0.1093	0.1001	0.1199	9.18	9.75	19.78	14	0.098
25	0.1332	0.1233	0.1456	8.05	9.26	18.08	13	0.092

*% Relative deviation between experimental observations and simulation results calculated as follows:

% Rel. Dev. = $$\frac{\left|\mathrm{Experimental\, observation }-\mathrm{ Simulation\, result }\right|}{\mathrm{Experimental\, observation}}$$ .

**% Relative deviation between simulation results and uniform mixing model calculated as follows:

% Rel. Dev. = $$\frac{\left|\mathrm{Simulation\, result }-\mathrm{ Analytical\, model\, prediction }\right|}{\mathrm{Simulation\, result}}$$.

***% Relative deviation between experimental results and uniform mixing model calculated as follows:

% Rel. Dev. = $$\frac{\left|\mathrm{Experimental\, observation }-\mathrm{ Analytical\, model\, prediction }\right|}{\mathrm{Experimental\, observation}}$$ .

****% Rel. SD: Relative standard deviation based on five sets of experiment results = $\frac{\mathrm{standard\, deviation}*100}{\mathrm{mean\, of\, the\, data}}$.

Our experiments were only performed at flow rates of up to 25 L min^−1^ but clear deviations remained (maximum of approximately 9%). The results showed that the CFD technique is a useful tool for estimating the distribution of ^220^Rn gas inside the chamber, and the simulation results also agreed well with the experimental observations at higher flow rates. In conclusion, our results clearly indicate that the ^220^Rn calibration chamber at RRI is suitable for calibrating SSNTDs.

## Conclusion

CFD is an inexpensive visualization tool that has been applied widely to solve health-related issues, such as predicting the activity concentrations of ^222^Rn and ^220^Rn gas inside confined areas. ^220^Rn gas is increasingly recognized as a potential source of radiation exposure in dwellings, so various organizations recommend assessing the long-term levels of indoor exposure (passive measurements). Obtaining accurate measurements requires determining the calibration factors for SSNTDs in order to predict the exact distribution of the ^220^Rn concentration inside a calibration chamber. Precise measurements are necessary to accurately assess ^222^Rn and ^220^Rn dose exposure. Therefore, in the present study, the distribution of the ^220^Rn concentration was simulated and predicted inside the calibration chamber at RRI using different configurations for the inlet and outlet under various flow rates. The facilities at RRI were suitable for producing and sustaining the required homogeneous concentrations of thoron. Initially, the results showed that the transmission factor (C_out_/C_in_) increased after increasing the flow rate under all five chamber configurations tested in this study. Besides that, in order to represent the non-uniformity of a concentration field, coefficient of variation (CoV) of the ^220^Rn concentration is also calculated. The positions of the inlet and outlet, and the flow rate were also identified as key factors that determined the distribution of the ^220^Rn concentration within a closed volume, thereby affecting the transmission factor for thoron.

The simulation results were also compared with the predictions obtained using the mixing model by defining the transmission factor (C_out_/C_in_), which can be used as a suitable indicator for checking the uniformity in terms of the distribution of the ^220^Rn concentration inside the chamber. In conclusion, our results clearly demonstrate that the ^220^Rn calibration chamber at RRI is suitable for calibrating SSNTDs. Moreover, the CFD-based predictions agreed well with the experimental and analytical results according to the relative deviations and close agreement between the corresponding transmission factors at higher flow rates. The result also suggests that, for the current physical configuration, special consideration should always be given to the flow rates, as it does not always satisfy the commonly used well-mixed hypothesis. This observation is very important for improving current calibration methods for measuring the activity concentration of ^220^Rn using passive monitors, in which well-mixed states are often assumed.

## Data Availability

The data sets used and/or analyzed during the current study are available from the corresponding author on request.
